# The Immunopathological and Histological Landscape of COVID-19-Mediated Lung Injury

**DOI:** 10.3390/ijms22020974

**Published:** 2021-01-19

**Authors:** Giovanni Zarrilli, Valentina Angerilli, Gianluca Businello, Marta Sbaraglia, Giulia Traverso, Francesco Fortarezza, Stefania Rizzo, Monica De Gaspari, Cristina Basso, Fiorella Calabrese, Angelo Paolo Dei Tos, Matteo Fassan

**Affiliations:** 1 Department of Medicine (DIMED), Surgical Pathology & Cytopathology Unit, University of Padua, 35121 Padua, Italy; giovanni.zarrilli@gmail.com (G.Z.); valentina.angerilli@gmail.com (V.A.); glc.businello@gmail.com (G.B.); marta.sbaraglia@aopd.veneto.it (M.S.); 2 Istituto Oncologico Veneto, IOV-IRCCS, 35128 Padua, Italy; me.giulia@gmail.com; 3 Department of Cardiac, Thoracic, Vascular Sciences and Public Health, University of Padua, 35121 Padua, Italy; francescofortarezza.md@gmail.com (F.F.); s.rizzo@unipd.it (S.R.); monica.deg1@gmail.com (M.D.G.); cristina.basso@unipd.it (C.B.); fiorella.calabrese@unipd.it (F.C.)

**Keywords:** SARS-CoV-2, COVID19, pathology, cytokine storm

## Abstract

A complete understanding of severe acute respiratory syndrome coronavirus 2 (SARS-CoV-2) physiopathology and related histopathologic lesions is necessary to improve treatment and outcome of coronavirus disease 2019 (COVID-19) patients. Many studies have focused on autopsy findings in COVID-19-related deaths to try and define any possible specific pattern. Histopathologic alterations are principally found within lungs and blood vessels, and these abnormalities also seem to have the highest clinical impact. Nevertheless, many of the morphological data collected so far are non-specific, fickle, and possibly associated with other co-existing factors. The aim of this minireview is to describe the main histopathological features related to COVID-19 and the mechanism known as “cytokine storm”.

## 1. Introduction

Severe acute respiratory syndrome coronavirus-2 (SARS-CoV-2) is responsible for the third large-scale epidemic related to coronaviruses, after SARS-CoV in 2002 and Middle East respiratory syndrome (MERS)-CoV in 2012. Coronavirus disease 2019 (COVID-19) was first reported in Wuhan, China, in December 2019, among a group of individuals presenting with pneumonia of unknown etiology [1].

The World Health Organization (WHO) declared COVID-19 a pandemic on 11 March, 2019. The number of confirmed cases as of 8 January 2021 is over 86 million, with almost two million deaths worldwide.

Coronaviruses are enveloped, positive-sense single-stranded large RNA viruses that infect humans as well as a wide range of animals. Their spherical morphology with a core shell and glycoprotein projections from their envelope gives them a “crown-like” appearance; therefore, they are termed coronaviruses [2]. They have the largest genome among all RNA viruses, typically ranging from 27 to 32 kB. Their four main structural genes encode the nucleocapsid protein (N), the spike protein (S), the small membrane protein (SM), and the membrane glycoprotein (M) [3].

Being transmitted mainly through direct or indirect respiratory tract exposure, SARS-CoV-2 uses angiotensin-converting enzyme 2 (ACE2) as its main receptor, which is broadly expressed in the respiratory epithelium and vascular endothelium [4].

Key mechanisms that may be fundamental in the pathophysiology of multi-organ injury caused by SARS-CoV-2 infection include: (i) direct virus-mediated cell damage; (ii) dysregulation of the renin–angiotensin–aldosterone system (RAAS) as a consequence of the downregulation of ACE2 related to viral entry; (iii) endothelial cell damage with subsequent inflammation and generation of a thrombotic milieu; and (iv) dysregulation of the immune response and cytokine release syndrome [5].

The aim of this minireview is to describe the main histopathological alterations and the immunopathological mechanisms underlying COVID-19, with particular attention to lung injury.

## 2. Histopathological Features of COVID-19

**Figure 1:**
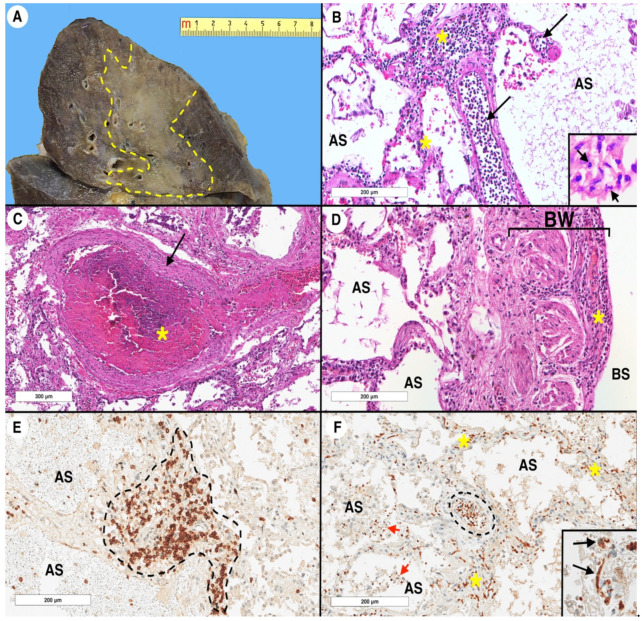
Coronavirus disease 2019 (COVID-19) lung pathology: a representative case. Specimens were obtained from a 69-year-old female who died from COVID-19 complications. The autopsy was carried out at the University Hospital of Padua. (**A**) Gross sample of the upper right lobe. The cut surface shows a large grayish area of consolidation, delimitated by the yellow dashed line. (**B**) At histology, the lung parenchyma shows interstitial vessels (black arrows) with numerous intraluminal inflammatory cells (i.e., margination), which also infiltrate the interstitial space (yellow asterisks) (hematoxylin and eosin stain, original magnification × 200). At a higher magnification, some polymorphonuclear leukocytes (black arrow) are evident (inset, hematoxylin and eosin stain, original magnification × 600). (**C**) A centrilobular arteriole (black arrow) completely occluded by a fibrin thrombus (yellow asterisk) (hematoxylin and eosin stain, original magnification × 100). (**D**) Bronchial wall section. The mucosa is completely denuded, with a significant inflammatory process in the submucosal layer (yellow asterisk) (hematoxylin and eosin stain, original magnification × 200). (**E**) A dense aggregate of lymphocytes with significant widening of the interstitial space (delimited by the dashed black line). The CD3 immunoreaction highlights that the inflammatory cells are mainly composed of T lymphocytes (CD3 immunoperoxidase staining, Novocastra, clone NCL-L-CD2-565, original magnification × 200). (**F**) Interleukin 6 (IL-6) immunohistochemical reaction that labeled the intravascular (dashed black line), interstitial (yellow asterisks), and alveolar (red arrows) lymphocytes. In the alveolar space, several macrophages (unstained cells with a large cytoplasm) are also evident (IL-6 immunoperoxidase staining, Abcam, clone ab9324, original magnification × 400). At a higher magnification, some endothelial cells express IL-6 (black arrows) and sometimes appear positive when injured. IL-6 immunoreaction corresponds to a cytoplasmic staining with perinuclear reinforcement (inset, IL-6 immunoperoxidase staining, original magnification × 400). AS: alveolar space; BW: bronchial wall; BS: bronchial space.

Our understanding of SARS-CoV-2 infection and its spreading among organs and tissues is rapidly evolving. All over the world, many studies have focused on autopsy findings in COVID-19-related deaths, trying to determine any possible specific pattern [6,7,8,9]. In this chapter, we will describe the most frequently reported and, in our opinion, most significant histological findings in SARS-CoV-2-infected patients, with a particular focus on pulmonary involvement (Figure 1).

### 2.1. Lung and Respiratory Tract Histopathology

The respiratory system is certainly the first target of investigation in COVID-19-related studies.

Many histopathological findings have been identified in autoptic specimens [10]. Nevertheless, it is important to notice that none of the described histological features is pathognomonic or highly specific [11], and future gene expression profiling studies should be performed on these specimens.

The most frequently reported morphological feature of COVID-19 is diffuse alveolar damage (DAD) [11,12,13,14,15,16,17,18,19,20,21,22,23,24,25,26,27], both in the exudative and in the proliferative phase (however, the latter phase does not always occur). The exudative phase lasts about 10 days on average, even though the case series displays great variability and is characterized by a variable degree of edema [12,13,14,15,16,18,21,24,28,29] and hyaline membrane formation [11,12,13,15,16,17,18,19,21,22,23,24,25,28]. On the other hand, the proliferative phase is characterized by fibroblast and myofibroblast proliferation, extracellular matrix deposition, and intra-alveolar fibrin accumulation [11,12,13,14,15,16,17,18,19,20,21,22,23,24,25,28,29,30]. Cases have been described in which the deposition of intra-alveolar fibrin was found to be the main histological feature of COVID-19 associated with organized pneumonia; these cases are called acute fibrinous and organizing pneumonia (AFOP) [31,32,33]. In the proliferative phase, occasional osseous metaplasia has also been found [30]; furthermore, microabscesses and Masson bodies have been described concurrently with the organizing phase [34]. The two phases of DAD are not synchronous events within the entire lung: they can take place with different timing in different areas of the pulmonary parenchyma [29]. DAD is a common finding in infective and non-infective pulmonary diseases and cannot be considered specific of COVID-19, even though it is among its most frequent morphological manifestations [11]. Alveolar structure and epithelium showed some non-specific alteration as well, the most frequently reported of which are squamous metaplasia [12,17,22,24,26], intra-alveolar hemorrhages [13,17,19,23,24,25,28], desquamation [13,17,24,28], and type II pneumocyte hyperplasia [12,14,17,19,20,21,22,24,25,26].

The inflammatory pattern was also evaluated: the detected immune cell population consisted predominantly of mononucleated cells, of which the most abundant fraction was composed of lymphocytes [11,12,13,15,16,17,18,19,20,21,22,25,28]. Nevertheless, neutrophils were found in a not negligible percentage of the cases, which varied between about 25% to 30% [12,13,16,19,20,22,24,25,27] and 50% [26]. Some authors believe that the neutrophilic component of the inflammatory background during COVID-19 is due to a bacterial superinfection [12]. This theory is partially supported by one of the autopsy series, in which the only patient who had neutrophilic inflammation was the only immunosuppressed one [18]. Inflammation in the form of lymphocytic infiltration has also been reported in other structures of the respiratory tract, such as bronchi [11,12,16,23], trachea [11,21] and pharynx [12]. Together with lymphocytic infiltration within the pulmonary syncytium, giant multinucleated cells were frequently detected [13,15,17,23,24,28,35]; nevertheless, these cells have been found to express the pulmonary differentiation marker TTF-1, rather than macrophage markers like CD68 [23].

Another interesting, yet clinically impractical, morphological feature to be evaluated in COVID-19 patients is the cytology of the pleural effusion; it has been shown that aggregates of dysmorphic mesothelial cells with enlarged nuclei and multinucleated syncytial cells can be found in pleural fluid cytoblock preparations from COVID-19 patients [15].

As already mentioned, pulmonary histology by itself is poorly specific for COVID-19 when compared with that of other pathologies; an interesting study has in fact proven that the pulmonary histological features of patients deceased because of COVID-19 or H1N1 influenza shared a common histological pattern of DAD and inflammatory infiltration [14].

### 2.2. Other Organs Histopathology

Blood vessel involvement through endotheliitis is one of the distinguishing features of COVID-19, and many histological endothelial modifications have been identified. A study by Ackermann and colleagues [14] summarized the three most important vascular features within COVID-19-affected lungs: (i) endothelial injury associated with intracellular localization of SARS-CoV-2 [14,24,36] with evidence of apoptosis induction [24,36]; (ii) vascular thrombosis within alveolar capillaries (arteries with a diameter of 1 to 2 mm and postcapillary venules also showing thrombotic features, though they were found to be less COVID-19-specific) [14]; (iii) intussusceptive angiogenesis, which caused the formation of distorted vessels [14]. Microthrombi within alveolar capillaries, precapillary arteries, and postcapillary venules were the most frequently reported vascular feature [15,16,18,19,21,24,27]. Endotheliitis is not exclusive to blood vessels within the lungs; it has in fact been reported in the vasculature of heart, kidney, and small bowel [36].

There are increasing clues pointing to the kidney as a potential target of COVID-19; direct viral infection and renin–angiotensin–aldosterone dysfunction have been suggested as major physiopathological mechanisms [37,38]. From a histopathological standpoint, a few remarkable features have been observed. Acute tubular damage [11,13,21,23,38], mostly reported within the proximal tract [24,39], together with vacuolar degeneration [23,39] are the most commonly detected features of renal involvement. Findings within glomeruli are, instead, less consistent throughout the literature: swollen glomerular cells [24] and capillaries obstructed by erythrocyte aggregates [24,39] have been described, while other authors suggest that glomeruli do not go through any severe injury [13]. An additional renal histopathological finding in COVID-19 patients is bacterial superinfection with associated acute pyelonephritis; nevertheless, this finding has been detected only in a small fraction of a series of autopsies (2 of 26 cases) [39].

In the COVID-19 landscape, testis have been proposed and studied both as a target for the infection and as a potential reservoir of the virus itself [24]. Histological manifestations of COVID-19 within testis are, nevertheless, unspecific, even though they seem to be rather consistent, if taking into consideration every degree of involvement. Edema has been reported [35] in association with interstitial inflammation, in which the main cellular components were lymphocytes and histiocytes [24,35]. Sertoli cells showed the most noticeable morphological alterations, as they were found to be detached from the tubular basement membrane and went through vacuolar degeneration [35]. Seminiferous tubules also showed histopathological alterations in the form of a non-specific injury which varied from mild to severe in grade [24,35].

Cardiac sampling in COVID-19-related autopsy series has been often performed, mostly to rule out myocarditis. The main histopathological findings suggesting cardiac involvement in COVID-19 patients are the presence of increased interstitial macrophages in a majority of the cases and multifocal lymphocytic myocarditis in a small fraction of the cases [40]. Scattered degenerated myocytes have been detected in association with scarce inflammation in the form of a lymphocytic infiltrate, often found to be adjacent to (not overlapping with) the degenerated cells [18,19]. Whether these morphological features represent myocarditis in its early form is not clear, nor are the clinical implications of these findings [11,18]; furthermore, the histopathological features of COVID-19 cardiac involvement have been described as focal and nonspecific, so that it is difficult to ascribe them to a direct damage caused by the virus to the myocardiocytes or to preexisting conditions [24].

Central nervous system (CNS) involvement in COVID-19 is a highly debated issue. Some authors described morphological alterations found in autopsy series of COVID-19-related deaths; the most significant described histopathological features were hypoxic injury [24], edema [24], hemorrhagic foci surrounded by damaged swollen axons [41], and perivenular localization of macrophages (CD68^+^) within areas of subcortical white matter pallor [41]. However, an interesting series of 18 COVID-19-related autopsies “[…] showed only hypoxic changes and did not show encephalitis or other specific brain changes referable to the virus. There was no cytoplasmic viral staining on immunohistochemical analysis” [42]. These contradictory findings between different studies are eloquent about the need for more data about the possible involvement of CNS in COVID-19 patients. 

Gastrointestinal clinical manifestations of COVID-19 are not uncommon; however, morphological alterations within the digestive tract are scant. Segmental stenosis and dilation of the small intestine were seen, in association with various degrees of mucosal degeneration [24]. Of note, ACE2 is abundantly present in enterocytes [43]. Viral particles have been detected in stool specimens, and diarrhea was reported in several infected patients [44].

The liver has been analyzed as well, in search of histopathological alterations attributable to COVID-19. However, also in this case, the morphological features were not unequivocal nor specific, and hepatic involvement could not be completely ruled out for at least a few reasons, i.e., (i) the evidence that liver expresses ACE2, predominantly on cholangiocytes [24]; (ii) liver function tests are found to be mildly or moderately altered in 4–10% of the patients [45]. Inflammation has been detected in the form of mild lymphocytic portal infiltrate [24,26] and aggregates of intraparenchymal lymphocytes [45]; together with inflammation, focal spots of necrosis and biliary plugs have been identified [24]. Another rather frequently reported histopathological feature within the liver of COVID-19 patients is steatosis, both micro- and macrovesicular [13,24,26]; nevertheless, it has been suggested that this finding is likely due to pre-existing conditions or possibly to drug toxicity [26,45].

One known clinical manifestation of COVID-19 is lymphocytopenia [13]; this evidence has driven interest toward the histopathological evaluation of the spleen and lymph nodes. The spleen has been found to show a congested pulp [13,27] with hemorrhagic spots [13]. Furthermore, the white pulp has been described as lacking lymphoid follicles [13] and depleted of CD8^+^ lymphocytes [23,27], while the plasma cellular component was found to be prominent [27]. Other architectural alterations such as interstitial vessels hyperplasia and fibrosis of the splenic sinus have been identified [13]. Within lymph nodes, one of the most interesting histopathological alterations that have been described is the presence of apoptotic lymphocytes; this finding is thought to be at least partially responsible for the aforementioned lymphocytopenia [13]. Other morphological findings regarding the lymph nodes are the preservation of lymphoid follicles (apparently antithetical with the spleen finding), the depletion of paracortical areas, the prominent plasma cellular component (this time in agreement with what observed in the spleen) and sinus histiocytosis [27].

Within the skin, two macroscopic manifestations have been described [7,46]: (i) viral exanthems (morbilliform rash, petechial rash co-existing with thrombocytopenia, erythematous-to-purpuric coalescing macules, widespread urticaria, and varicella-like vesicles); and (ii) vasculopathy-related skin manifestations (peripheral cyanosis with bullae and dry gangrene, transient unilateral livedo reticularis, and red papules on fingers resembling chilblains). In biopsy samples, histopathological analysis revealed superficial and deep perivascular dermatitis, perivascular lymphocytic infiltration, focal acantholytic suprabasal clefts, dyskeratotic and ballooning herpes-like keratinocytes, necrosis of keratinocytes, mucin deposition in the dermis and hypodermis, and nests of Langerhans cells within the epidermis [7,46]. Other described features are thrombus formation and extravasation of erythrocytes from mid-dermis blood vessels [47,48].

## 3. Cytokine Storm

“Cytokine storm” (CS) is an evocative term which is increasingly used by both scientists and mass media to describe an uncontrolled and generalized inflammatory response [49]. A “cytokine storm” has been defined by Cron and Behrens as an activation cascade of auto-amplifying cytokine production due to an unregulated host immune response to different triggers [50], though this description is not universally accepted.

This process has been advocated to explain the pathophysiological events taking place in patients with severe forms of COVID-19, in an attempt to relate the high levels of proinflammatory cytokines to life-threatening conditions (i.e., lung injury, acute respiratory distress syndrome (ARDS), multiple-organ failure (MOF)) affecting these patients [51]. Notably, a cytokine storm has been theorized and described in other viral infections, such as influenza (by H5N1 and H1N1 virus alike), SARS, and MERS [52].

Some authors appeal to caution in using the term “cytokine storm”, since no solid data linking CS to COVID-19 are available yet. In their opinion, the manifestations of elevated circulating mediators are probably due to endothelial dysfunction and systemic inflammation and could be explained by the systemic inflammatory response syndrome (SIRS), with fever, tachycardia, tachypnea, and hypotension [53]. Moreover, the true nature of the so-called cytokine storm is not completely understood yet [49].

Leaving aside the dispute on whether a “cytokine storm” occurs or is just a simplistic assumption to describe a more complex process, many studies suggest that there is a kind of direct proportionality between proinflammatory cytokines levels and the severity of symptoms caused by COVID-19. In particular, higher levels of cytokines have been documented in COVID-19 patients with severe illness compared to those with a moderate one [54,55,56,57,58]; furthermore, the enhanced level of cytokines seems to be associated with a lowered T lymphocytes count [59]. Another fact that somewhat supports the occurrence of a cytokine storm as a true entity is that patients with increased levels of cytokines have been found to have a poorer prognosis [50]. Additionally, the levels of inflammatory indices seem to be related to disease severity: COVID-19 patients admitted to intensive care unit (ICU) usually present higher levels of white blood cells, neutrophils, procalcitonin, and C-reactive protein (CRP) compared to non-ICU patients [60,61].

The interaction of SARS-CoV2 proteins with its receptor ACE2 seems to be one of the earliest events in the pathogenesis of the cytokine storm [51,62]. This interaction is thought to lead to the entrance of SARS-CoV-2 within the respiratory epithelial cells; lung infection leads to the activation of alveolar macrophages and lung epithelial cells that will in turn produce several inflammatory cytokines, such as IL-1β, IL-2, IL-6, IL-7, IL-8, IL-10, Tumor necrosis factor α (TNF-α), Granulocyte-macrophage colony-stimulating factor (GM-CSF) and Interferon-gamma induced protein 10 (IP-10) [63].

High levels of IL-1β, IL-8, TNF-α can be found in the bronchoalveolar lavage fluid (BALF) from patients with ARDS [64]. IL-8 promotes neutrophil survival and recruitment to the lungs [65]. TNF-α is responsible for the apoptosis of lung epithelial and endothelial cells and the subsequent impairment of the lung microvascular and alveolar epithelial cell barrier resulting in vascular leakage and alveolar edema [66]. GM-CSF has the key role of mediating intercellular communications between Th1 cells and CD14^+^ CD16^+^ monocytes, which are accountable for the induction and amplification of tissue infiltration by macrophages. Of note, high numbers of CD14^+^ CD16^+^ monocytes are detectable in COVID-19 patients with severe lung injury [67]. Furthermore, exerting a strong chemotactic action, IP-10 promotes the migration of T lymphocytes, monocytes, and natural killer cells to the lungs [68]. 

IL-1β, IL-6, TNF-α increase the expression of cell adhesion molecules (CAMs) and vascular endothelial growth factor (VEGF) in the lung endothelium upon tissue injury, thus causing the destruction of the lung glycocalyx and increasing the permeability of the endothelium, allowing the virus to reach other organs that express ACE2. After entering the bloodstream, the pro-inflammatory cytokines stimulate the bone marrow to produce and release immature granulocytes, that return to the lungs and further increase lung inflammation, leading to ARDS [69]. Furthermore, activated monocytes express tissue factor and phosphatidylserine on their surface and initiate coagulation. However, while healthy endothelial cells maintain their anti-thrombogenicity, damaged endothelial cells become procoagulant following the disruption of the glycocalyx [70].

The presence of profibrotic factors such as transforming growth factor β (TGF-β) within the cytokine storm could promote impaired tissue remodeling and lung fibrosis [67].

Early extensive lung epithelial and endothelial damage plays a crucial role in creating an inflammatory loop. Cell death leads to the release of damage-associated molecular patterns (DAMPs), such as high-mobility group box 1 (HMGB1) and heat shock proteins (HSPs), which bind to pattern recognition receptors (PRRs), subsequently activating inflammatory signaling [71]. Moreover, it appears that there is an interplay between the oxidative stress generated within the inflammatory state and the cytokine storm that sustains and worsens the tissue injury, by exacerbating hypoxia. The oxidative stress also plays a role in the pathogenesis of COVID-19-related coagulopathy [72].

Another pivotal proinflammatory element in the physiopathology of COVID-19 is represented by the RAAS [51]. As said before, SARS-CoV-2 enters the respiratory epithelial cells through its interaction with ACE2. This interaction results in the internalization of the receptor itself and thus in its downregulation [51,56]. Reduced ACE2 expression enhances vascular permeability, causes lung edema, increases neutrophil accumulation, and therefore diminishes lung function. 

The downregulation of ACE2 also leads to a reduced transformation of angiotensin II into angiotensin-(1–7) and thus, to a hyperactivity of the angiotensin II/ATR1 axis [51,62]. Among other functions, the binding of angiotensin II to ATR1 is responsible for the activation of NF-κB, which is thought to have a pivotal role in COVID-19-related cytokine storm [51]. AT1R can activate ADAM metallopeptidase domain 17 (ADAM17), which is in turn responsible for the degradation of interleukin 6 receptor subunit α (IL-6Rα) into its soluble configuration sIL-6Rα [73]. Circulating sIL-6Rα can bind its ligand IL-6, causing the intracellular activation of the JAK/STAT3 pathway, which is a well-known trigger of NF-κB [73]. NF-κB is among the most important checkpoints involved in COVID-19-related proinflammatory events [62,74] and it is a pivotal protein complex involved in immune regulation [75].

Activated NF-κB leads to the production of many proinflammatory cytokines, among which, IL-6. This process, in which STAT3 and NF-κB are simultaneously activated, is called “IL-6 amplifier” [73] and it is well studied in other pathological conditions, such as major histocompatibility complex (MHC) class II-associated autoimmune diseases [76] and transplantation rejection [77,78]. In the case of COVID-19, NF-κB is activated via angiotensin II/AT1R, and STAT3 via IL-6/sIL-6rα/gp130. This results in a self-powered positive feedback loop of NF-κB activation with consequent production of cytokines, where IL-6 plays the most significant role [73].

In light of the previously described molecular mechanisms, from a pathologic standpoint, the excessive production of angiotensin II is responsible for pulmonary vasoconstriction, cytokine-induced organ damage, and epithelial cell apoptosis, while unopposed RAAS activation via angiotensin II/AT1R has been linked to inflammation, increased vascular permeability, and severe lung injury [79]. Furthermore, ACE downregulation results in decreased production of angiotensin-(1–7) that act through Mas receptor, exerting anti-inflammatory, anti-fibrotic, and vasodilatory effects [80].

## 4. Discussion

COVID-19 is a major health issue, responsible for more than a million deaths worldwide. Moreover, this pandemic has caused all non-urgent medical procedures to be postponed [81].

A complete understanding of the underlying immunopathological mechanisms of COVID-19 and its histopathological features is pivotal to help clinicians to improve disease treatment and outcome. Histopathological alterations are mostly found within lungs and blood vessels and seem to be the ones with the highest clinical impact; within other organs, as said above, the morphologic landscape of COVID-19 is poorly understood at best and inconsistent in many cases. Thus, more studies and evidence on tissue samples are required to establish whether other organs and tissues are indeed affected by COVID-19 and, possibly, to define the degree of their involvement.

The immunopathological scenario of COVID-19 is poorly characterized as well. The so-called cytokine storm has been proposed as a mechanism to explain the massive inflammatory response which is thought to be behind the most severe manifestations of the disease. Even though many of the proposed molecular mechanisms are intriguing, the precise pathways involved in COVID-19 are far from being completely clarified, and their role as main players or as collateral phenomena still needs to be established. 

To summarize, to date, the available knowledge on COVID-19 histopathology and immunopathology is still partial and poorly specific; therefore, more studies are needed to gather data that may prove useful for clinicians to improve the therapeutic approach to this disease. 

## Data Availability

Data are available upon request.
